# Relevance of self-rated health level and mental health in Korean adolescents

**DOI:** 10.3389/fpubh.2025.1526127

**Published:** 2025-05-14

**Authors:** Yunjeong Kim, Joohee Shim

**Affiliations:** ^1^Department of Healthcare Administration, Yeungnam University College, Daegu, Republic of Korea; ^2^College of Nursing, Yeungnam University College, Daegu, Republic of Korea

**Keywords:** adolescent, health behavior, mental health, self-rated health, physical activity

## Abstract

**Introduction:**

In this study, we explored the impact of mental health on self-rated health of Korean adolescents. By examining the relationship between self-assessed health and mental-health issues, such as anxiety and depression, this study sought to inform strategies for enhancing health education and school-based interventions.

**Methods:**

Secondary data from the Korea National Health and Nutrition Examination Survey were analyzed, with a sample comprising 418 middle and high school students. The study examined general characteristics of the sample and the correlations between subjective health status and mental-health indicators and between subjective health status and physical activity levels. Frequency and cross-tabulation analyses were performed to explore these relationships. Logistic regression analysis was conducted to identify factors influencing subjective health status. Statistical significance was set at *p* < 0.05.

**Results:**

Associations were noted between subjective health status and mental-health indicators, including stress recognition (*p* < 0.05), suicidal ideation in the past year (*p* < 0.001), suicide planning in the past year (*p* < 0.001), suicide attempts in the past year (*p* < 0.001), and counseling for mental-health problems in the past year (*p* < 0.005). Additionally, subjective health status was correlated with the following physical activity metrics: average daily hours spent seated (*p* < 0.05) and engagement in physical activities lasting 1 h or more per day (*p* < 0.1). Several factors were identified to influence subjective health status including the frequency of breakfast consumption per week (*p* < 0.05) and suicidal ideation in the past 1 year (*p* < 0.05).

**Discussion:**

The study findings highlight the considerable effects of mental health and health behaviors on adolescents’ subjective health status. Thus, addressing school health should go beyond promoting physical health alone. An environment that supports adolescents’ mental and social well-being, enabling them to grow into healthy adults, is necessary.

## 1 Introduction

Adolescence represents the transitional phase between childhood and adulthood, encompassing elements of biological maturation and evolving social roles, which have changed over the past century (1). While no universally accepted definition of adolescence exists, the World Health Organization (WHO) defines adolescence as spanning ages of 10–19 (2). In Korea, the definition varies depending on legal standards. This developmental stage is marked by rapid psychosocial and cognitive growth (3), shaping awareness of lifestyle and health choices, such as physical activity, diet, alcohol consumption, and smoking (4). Therefore, providing information and opportunities for healthy growth and development is essential, and expanding these opportunities is crucial in addressing the specific needs and rights of adolescents (2). However, due to changes in social norms, increased urbanization, and shifting trends in premarital sexual behavior, the physical and social landscape in which adolescents are growing is also evolving. As a result, there is a greater need than ever for research on adolescent health, as well as the establishment of programs and policies to address these changes (5). With increasing significance of adolescent health, the demand for representative data to establish and evaluate national-level adolescent policies has intensified. In South Korea, data is continuously collected at the national level to understand the various environmental factors affecting the health and growth of children and adolescents, with the aim of supporting their healthy development (6). Among these, the National Health and Nutrition Survey is conducted annually, surveying 25 households in 192 regions. The survey is categorized by life cycle stages, with separate analyses for children (ages 1–11), adolescents (ages 12–18), and adults (ages 19 and older) (7).

In South Korea, adolescents have shown decreasing levels of happiness and life satisfaction, largely owing to the pressures of an educational system centered around college entrance examinations and a highly competitive academic environment. Their reported life satisfaction is lower than that of other age groups within South Korea and ranks below that of adolescents in other countries (2–5). According to the Organization for Economic Co-operation and Development (OECD), only 32.0% of South Koreans aged 15 years and above considered their health to be good, which is less than half of the OECD average of 67.9%. Meanwhile, 17.2% reported poor health, which is the highest proportion among OECD countries and nearly double the average of 9.0% (8).

Subjective health perception refers to an individual’s self-assessed health status and serves as an indicator of well-being, incorporating not only the absence of physical illness but also mental and social wellness. Liang (9) defined health status in three dimensions: medical, functional, and self-assessed. Among these dimensions, self-rated health assessment is widely used as a universal tool for measuring overall health, serving as a key health indicator for individuals across all age groups and is particularly relevant from adolescence to old age (9, 10). With increasing recognition of subjective well-being in health, numerous studies on self-rated health have been conducted. However, most of these studies have focused on older individuals (11–13), while research on adolescents has been relatively limited, as adolescents are generally perceived as medically healthy (3). Subjective health perception often differs from objective medical findings, and this discrepancy is particularly evident in adolescents. Unlike adults, adolescents tend to evaluate their health based on overall functionality and well-being rather than the presence or absence of physical illness (4). Health determinants are multifaceted encompassing personal, social, ecological, pathological, political, and geographical factors. Research has examined subjective health status from various perspectives, highlighting its importance as a reflection of individuals’ psychological characteristics (4, 14).

Anxiety and depression are the most common mental-health problems experienced by adolescents. While depression manifests in late adolescence or adulthood, anxiety and depression often occur simultaneously in adolescent populations (15). Previous studies reported that mental health-related problems, such as depression and anxiety, are associated with suicidal ideation, and suicide attempts are associated with depression and behavioral disorders (16, 17). In recent years, the world has undergone significant upheaval. The coronavirus disease (COVID-19) pandemic, which began in 2019, has had a profound negative impact on adolescent mental health, leading to high levels of psychological distress (18). A recent study involving 80,000 adolescents worldwide reported that during the pandemic, depression and anxiety symptoms doubled, with 20% of adolescents experiencing severe anxiety symptoms (19). Additionally, a meta-analysis observed suicidal ideation among adolescents increased by 15% during 2020–2021 compared to the pre-pandemic period (20). Thus, research on the correlation between adolescents’ subjective health status and their mental health is an urgent need. Despite evidence linking subjective health status to mental health, including factors like stress, local research on this topic is limited. In particular, since transition through adolescence is inevitable, health awareness and appropriate action are likely to be more effective than other periods (21).

In this study, we aimed to establish the correlation between adolescents’ subjective health status and their mental health, providing data that can inform policies to improve adolescent health. The study findings can also serve as foundational data to address health inequalities among adolescents and to guide the development of physical and mental-health programs at the school level.

The hypotheses of this study were as follows:

1. There is a significant association between Korean adolescents’ subjective health status and their mental health.
2. There is a significant association between Korean adolescents’ subjective health status and their level of physical activity.
3. Korean adolescents’ subjective health status is influenced by socioeconomic factors, physical activity levels, and mental-health indicators.

## 2 Materials and methods

This study utilized data from the 2011 Korea National Health and Nutrition Examination Survey (KNHANES), a nationwide cross-sectional survey conducted by the Korea Disease Control and Prevention Agency (KDCA). KNHANES employs a complex, stratified, multistage probability sampling design and uses standardized, validated questionnaires to assess various health indicators. The KNHNES consists of health, medical examination, and nutrition surveys; however, this study used only data from the health survey for analysis. The 2011 survey was conducted in accordance with the Declaration of Helsinki, and although it was exempted from institutional review board (IRB) approval at the time of data collection, ethical standards were maintained by the KDCA, and informed consent was obtained from all participants. The dataset is publicly available for secondary analysis. Data access was obtained through a formal request and approval from the relevant agency.

For the study sample, individuals in the 12–18-year age group without missing data on the selected variables were chosen, and weighted data were applied. The final analysis included 418 participants. Key health-related variables among adolescents, including educational stage, sex, income quintile, health insurance type, degree of stress recognition, sleep duration per week, frequency of breakfast consumption per week, drinking and smoking experiences, and subjective health status, were identified and used in the analysis.

### 2.1 Research materials and participants

The data analyzed were collected from the third year (2021) of the eight-phase of the KNHNES conducted jointly by the Ministry of Health and Welfare and the Korea Disease Control and Prevention Agency. This study is a secondary analysis, and the researchers proceeded after officially requesting and receiving approval to use the data. The researchers proceeded with the study after making an official data request to the relevant agencies and receiving approval. The KNHNES consists of health questionnaire surveys, medical examinations, and nutrition surveys. This study utilized only data from the health questionnaire survey for analysis.

Informed consent was obtained from all participants and their legal guardians before data collection. For sensitive topics, specific protocols were in place to handle potential disclosures of harm or risk, including immediate referral to appropriate support services if necessary. All collected data were anonymized and stored securely in accordance with strict data protection protocols. Personal information was ethically anonymized, with confidentiality maintained through the use of unidentifiable serial numbers.

Data collection spanned over 1 year, from January to December 2021. Data collection was conducted using mobile examination centers (MECs), which provided a private and secure environment for participants. Adolescents completed the individual component of the health interview questionnaire, including questions on sensitive topics such as mental health and suicidal ideation, via self-administration in a private area within the MEC. This approach ensured privacy and anonymity for the adolescent respondents.

**Figure 1:**
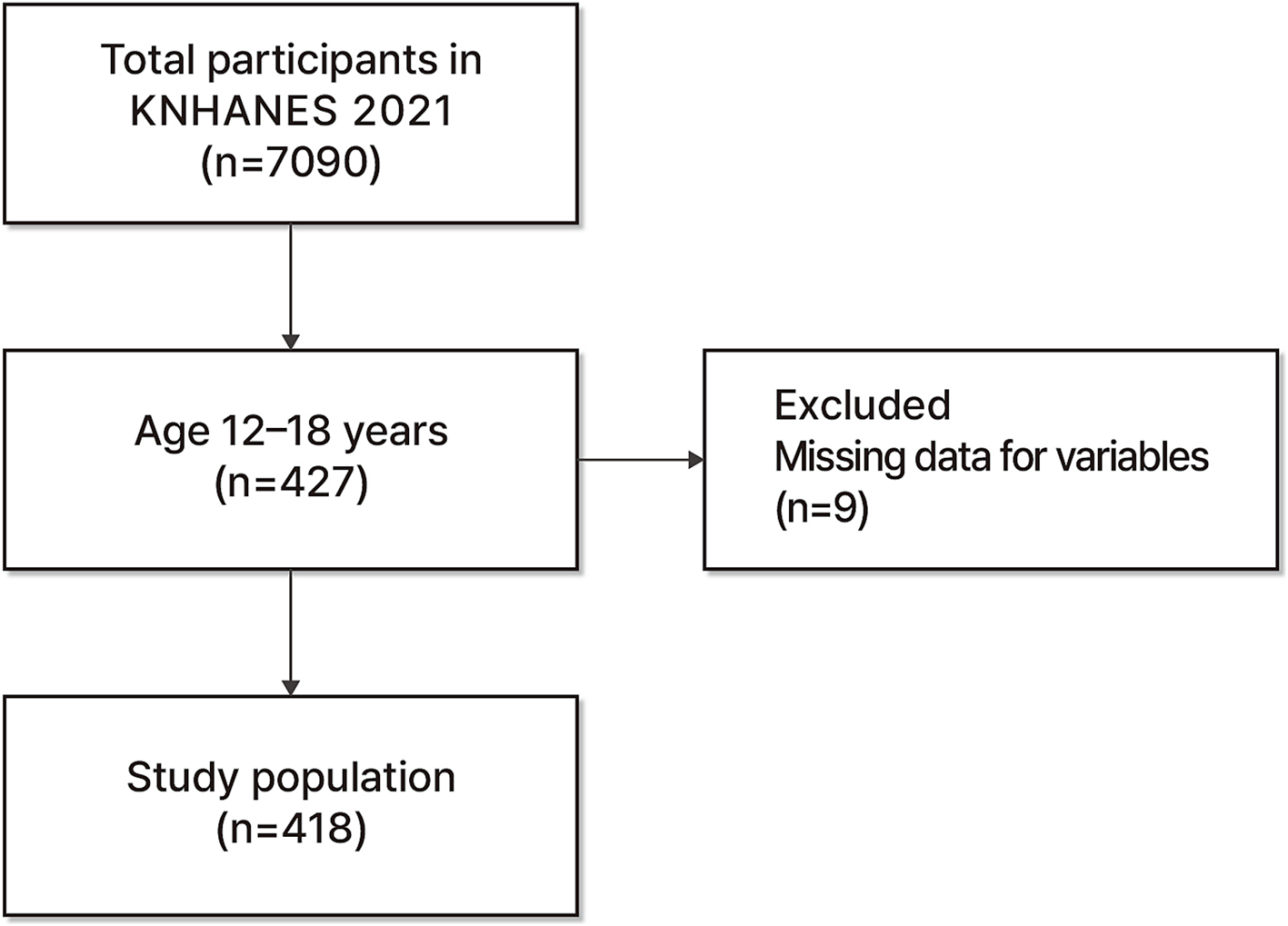
Selection of participants.

For this study, data were collected using a rolling sample survey approach with sampling hierarchized based on city/province, neighborhood/town/township, and housing type. Although the raw data included the entire population aged 1 year or older, only data from adolescents were included in this analysis, with data from other age groups excluded. Out of the total 7,090 cases, 427 cases corresponding to the 12–18-year age group were selected as the sample population. After excluding 9 cases with missing data, a total of 418 cases were included in the final analysis (Figure 1). In accordance with KDCA data analysis guidelines, the researchers did not manipulate the raw data.

### 2.2 Data collection

The KNHNES is a legally initiated survey under the National Health Promotion Act, designed to assess health behaviors, prevalence of chronic illnesses, and dietary intake. Each year, 192 households are selected for participation, and across the 3 years of the eight phase (2019–2020), a total of 576 households were included. From these households, approximately 10,000 individuals aged 1 year or older were selected as survey participants. Based on life cycle characteristics, participants were categorized as children, adolescents, or adults, and survey items were tailored to suit each group. Beginning with the fourth phase, the KNHNES adopted a year-round survey system and implemented a rolling sample method. This approach allows each of the 3 years within a phase to consist of three independent rolling samples, providing probability samples that are representative of the entire nation. Medical examination surveys were conducted using direct measurements and specimen analyses, while health surveys were performed through interview surveys and self-administered surveys during visits to selected households. Sensitive topics such as mental-health indicators were handled using strict ethical protocols to ensure participant safety and confidentiality throughout the process.

### 2.3 Study variables

Variables were selected based on previous research by Prasad et al. (14), Zhao et al. (22), and Han and Park (17). Independent variables included educational stage, sex, income quintile, health insurance type, degree of stress recognition, sleep duration per week, frequency of breakfast consumption per week, and mental-health indicators such as presence of prolonged depression (lasting 2 weeks or more), suicidal ideation, suicide planning, and suicide attempts within the past year, as well as mental-health counseling sought within the past year. Additional variables include average daily hours spent seated, engagement in physical activities lasting 1 h or more per day, and the frequency of strength training sessions per week. The dependent variable was participants’ subjective health status.

#### 2.3.1 Sociodemographic characteristics

Sociodemographic characteristics were assessed using variables such as educational stage, sex, income quintile, and health insurance type. For educational stage, respondents who answered either “Middle school” or “High school” to the question, “Up to which level did you go to school? Or are you attending school?” and indicated “Enrolled” in response to the question, “Did you complete that school? Please tell us whether or not you graduated,” were classified into the categories “Middle school” or “High school.” Household income quintiles were classified as follows: responses of “Lower,” “Lower middle,” “Middle,” “Upper middle,” and “Upper” were categorized into “Quintile 1,” “Quintile 2,” “Quintile 3,” “Quintile 4,” and “Quintile 5,” respectively. In this study, these classifications have been adapted to align with the U.S. income quintiles, which are defined as follows: Quintile 1, $0 to $31,462; Quintile 2, $31,463 to $59,312; Quintile 3, $59,313 to $96,782 L; Quintile 4, $96,783 to $157,722; Quintile 5, $157,723 and above. As of January 23, 2025, $157,723 is equivalent to approximately €143,385 or £122,824; notably, the actual amounts may vary due to exchange rate fluctuations. Regarding health insurance type, respondents who answered “Locally provided National Health Insurance,” “Employer-provided National Health Insurance,” or “Medical aid” to the question, “Which health insurance do you subscribe to?” were included in the corresponding health insurance categories.

#### 2.3.2 Health behavior

Health behavior variables included sleep duration per week and the frequency of breakfast consumption per week. For sleep duration per week, responses to the question, “During the week, at what time do you usually go to bed and get up?” were classified into three categories: Fewer than 7, 7–8, and 9 h or more. Regarding breakfast frequency, responses to the question, “In the past year, how many times did you have breakfast per week?” were categorized as 0, 1–2, 3–4, and 5 days or more.

#### 2.3.3 Mental health

To assess participants’ mental health, variables included stress recognition in daily life, prolonged depression lasting 2 weeks or more, suicidal ideation within the past year, suicide planning in the past year, and suicide attempts in the past year. For stress recognition, participants were asked, “In your daily life, how much stress do you feel?” Responses categorized as “Very much” and “Much” were combined under “Much,” while “Some” and “Hardly” were grouped as “Some.” Responses to questions regarding depression and suicidal ideation were classified as “Yes” and “No.” The survey questions were designed as follows: Prolonged depression for 2 weeks or more was measured by asking, “In the past year, have you experienced sadness or despair continuously for 2 weeks or more, to an extent that it interfered with your daily life?” Suicidal ideation over the past year was assessed by asking, “In the past year, have you seriously considered suicide?” Suicide planning within the past year was addressed by asking, “In the past year, have you seriously planned suicide?” Lastly, suicide attempts in the past year were assessed with the question, “In the past year, have you actually attempted suicide?”

#### 2.3.4 Subjective health status

To evaluate participants’ subjective health status, the question “How do you usually feel about your health?” was included, with response options of “Very good,” “Good,” “Average,” “Bad,” and “Very bad.” To simplify analysis based on the distribution of responses, these were reclassified as follows: “Very good” and “Good” were combined as “Good,” “Average” remained as “Average,” and “Bad” and “Very bad” were grouped as “Bad.”

### 2.4 Data analysis

To determine the correlation between adolescents’ subjective health status and mental health, we analyzed data using IBM SPSS Statistics for Windows, version 24.0 (IBM Corp., Armonk, NY, United States). Using proportional stratified sampling (a systematic sampling technique), weighted values provided by the KDCA were applied in our analysis. We evaluated the general characteristics, correlations between subjective health status and mental health, and the correlation between subjective health status and physical activity levels. We conducted frequency and cross-tabulation analyses. In addition, logistic regression analysis was conducted to identify factors affecting subjective health status. For all tests, the significance level was set at *p* < 0.05.

### 2.5 Ethical approval

This study was a government-approved statistical survey (Approval No. 117002), conducted in accordance with the National Health Promotion Act. KNHNES data were obtained after receiving official approval from the Korea Disease Control and Prevention Agency. Data collection was facilitated using mobile examination centers (MECs), which provided a private and secure environment for participants. Adolescents completed the individual component of the health interview questionnaire, including questions on sensitive topics such as mental health and suicidal ideation, via self-administration in a private area within the MEC. This approach ensured privacy and anonymity for the adolescent respondents. Informed consent was obtained from all participants and their legal guardians before data collection. For sensitive topics, specific protocols were in place to handle potential disclosures of harm or risk, including immediate referral to appropriate support services if necessary. All collected data were anonymized and stored securely in accordance with strict data protection protocols. Personal information was ethically anonymized, with confidentiality maintained through the use of unidentifiable serial numbers. The Institutional Review Board of Yeungnam University College (No. 2-7008156-AB-N-01-A-2022-005) granted approval for this study and waived the requirement for further ethical deliberation owing to the secondary nature of the data analysis and robust ethical procedures already in place for the original data collection.

## 3 Results

### 3.1 General characteristics based on adolescents’ subjective health status

Adolescent characteristics were examined based on subjective health status, and the results are as follows (Table 1). Among the total of 418 participants, subjective health status was reported as Good (59.3%), Average (35.9%), or Bad (4.8%), with “Good” being the most frequently reported. The most common responses for each characteristic were as follows: Middle school (55.5%) for educational stage, Male (56.0%) for sex, Quintile 4 (28.5%) for income quintile, Employer-provided health insurance (74.4%) for health insurance type, A little (56.0%) for the degree of stress recognition, 7–8 h (51.2%) for sleep duration per week, 5 days or more (44.1%) for frequency of breakfast consumption per week. Significant differences were observed in income quintile (*p* = 0.005), degree of stress recognition (*p* = 0.050), frequency of breakfast consumption per week (*p* = 0.003).

**Table 1:** General characteristics based on adolescents’ subjective health status.

Variable	N	Subjective health status			χ^2^ (*p*)
		Good	Average	Bad	
Educational stage					
Middle school	232(55.5)	142(57.3)	82(54.7)	8(40.0)	2.298
High school	186(44.5)	106(42.7)	68(45.3)	12(60.0)	
Sex					
Male	234(56.0)	146(58.9)	77(51.3)	11(55.0)	2.163
Female	184(44.0)	102(41.1)	73(48.7)	9(45.0)	
Income quintile					
Quintile 1	21(5.0)	6(2.4)	10(6.7)	5(25.0)	22.112**
Quintile 2	66(15.8)	38(15.3)	25(16.7)	3(15.0)	
Quintile 3	115(27.5)	71(28.6)	40(26.7)	4(20.0)	
Quintile 4	119(28.5)	71(28.6)	44(29.3)	4(20.0)	
Quintile 5	97(23.2)	62(25.1)	31(20.6)	4(20.0)	
Health insurance type					
Locally provided national health insurance	89(21.3)	48(19.4)	35(23.3)	6(30.0)	9.981*
Employer-provided national health insurance	311(74.4)	193(77.8)	107(71.3)	11(55.0)	
Medical aid	18(4.3)	7(2.8)	8(5.4)	3(15.0)	
Subjective body shape					
Skinny	99(23.7)	62(25.0)	32(21.3)	5(25.0)	18.984**
Normal	175(41.9)	120(48.4)	51(34.0)	4(20.0)	
Obese	144(34.4)	66(26.6)	67(44.7)	11(55.0)	
Degree of stress recognition					
Very much	19(4.5)	9(3.6)	7(4.7)	3(15.0)	12.563*
Much	85(20.3)	46(18.5)	32(21.3)	7(35.0)	
Some	234(56.0)	140(56.5)	84(56.0)	10(50.0)	
Hardly	80(19.1)	53(21.4)	27(18.0)	–	
Sleep duration per week					
<7 h	146(34.9)	83(33.5)	55(36.7)	8(40.0)	1.406
7–8 h	214(51.2)	127(51.2)	77(51.3)	10(50.0)	
9 h or more	58(13.9)	38(15.3)	18(12.0)	2(10.0)	
Frequency of breakfast consumption per week					
0 days	72(18.1)	35(14.9)	36(25.0)	1(5.6)	19.749**
1–2 days	72(18.1)	38(16.2)	27(18.8)	7(38.9)	
3–4 days	78(19.6)	43(18.3)	29(20.1)	6(33.3)	
5 days or more	175(44.1)	119(50.6)	52(36.1)	4(22.2)	
Total	418(100.0)	248(59.3)	150(35.9)	20(4.8)	

*p < 0.1, **p < 0.01, ***p < 0.001.

When subjective health status was reported as Good, the most frequent characteristics included Middle school for educational stage, Male for sex, Quintile 4 for income quintile, Employer-provided health insurance for health insurance type, 7–8 h for sleep duration per week, 5 days or more for frequency of breakfast consumption per week.

### 3.2 Correlation between adolescents’ subjective health status and mental health

The correlation between adolescents’ subjective health status and mental health was examined, and the results are as follows (Table 2). The most common responses were as follows: “A little” (75.1%) for stress recognition, “None” (94.3%) for episodes of persistent depression lasting 2 weeks or more, “None” (97.8%) for suicidal ideation in the past year, “None” (99.3%) for suicide planning, “None” (99.3%) for suicide attempts, and “None” (97.1%) for experiences of counseling for mental-health problems. Stress recognition (*p* = 0.020), suicidal ideation in the past year (*p* = 0.000), suicide planning in the past year (*p* = 0.000), suicide attempts in the past year (*p* = 0.000), and counseling for mental-health problems (*p* = 0.004) exhibited statistically significant differences.

**Table 2:** Correlation between adolescents’ subjective health status and mental health.

Variable	N	Subjective health status			χ^2^ (*p*)
		Good	Average	Bad	
Degree of stress recognition					
Some	314(75.1)	193(77.8)	111(74.0)	10(50.0)	7.822*
Much	104(24.9)	55(22.2)	39(26.0)	10(50.0)	
Prolonged depression for 2 weeks or more					
No	394(94.3)	235(94.8)	142(94.7)	17(85.0)	3.328
Yes	24(5.7)	13(5.2)	8(5.3)	3(15.0)	
Suicidal ideation in the past year					
No	409(97.8)	24(99.6)	145(96.7)	17(85.0)	20.264***
Yes	9(2.2)	1(0.4)	5(3.3)	3(15.0)	
Suicide planning in the past year					
No	415(99.3)	248(100.0)	149(99.3)	18(90.0)	25.982***
Yes	3(0.7)	–	1(0.7)	2(10.0)	
Suicide attempts in the past year					
No	415(99.3)	248(100.0)	149(99.3)	18(90.0)	25.982***
Yes	3(0.7)	–	1(0.7)	2(10.0)	
Counseling for mental-health problems in the past year					
No	406(97.1)	243(98.0)	146(97.3)	17(85.0)	11.224**
Yes	12(2.9)	5(2.0)	4(2.7)	3(15.0)	
Total	418(100.0)	248(59.3)	150(35.9)	20(4.8)	

*p < 0.1, **p < 0.01, ***p < 0.001.

When adolescents reported good subjective health status, the most frequent responses were as follows: A little (77.8%) for stress recognition, None (94.8%) for episodes of persistent depression lasting 2 weeks or more, None (99.6%) for suicidal ideation, None (100.0%) for suicide planning, None (100.0%) for suicide attempts, and None (98.0%) for mental-health counseling. For those with an average subjective health status, the most frequent responses included: A little (74.0%) for stress recognition, None (94.7%) for persistent depression episodes lasting 2 weeks or more, None (96.7%) for suicidal ideation, None (99.3%) for suicide planning, None (99.3%) for suicide attempts, and None (97.3%) for counseling for mental-health problems. When adolescents’ subjective health status was bad, the most common responses were as follows: A little (50.0%) and A lot (50.0%) for stress recognition, None (85.0%) for depression episodes lasting 2 weeks or more, None (85.0%) for suicidal ideation, None (90.0%) for suicide planning, None (90.0%) for suicide attempts, and None (85.0%) for counseling for mental-health problems.

### 3.3 Correlation between adolescents’ subjective health status and physical activities

The correlation between adolescents’ subjective health status and physical activity was examined, and the results are as follows (Table 3). The most frequent responses were as follows: 9–10 h (30.6%) for the average daily hours spent seated, None (63.2%) for engagement in physical activities lasting 1 h or more per day, and 1 day (60.3%) for the frequency of strength training sessions per week. The average daily hours spent seated (*p* = 0.010) and engagement in physical activities lasting 1 h or more per day (*p* = 0.050) exhibited statistically significant differences. When adolescents’ subjective health status was good, the most frequent responses were 11–12 h and 13 h or more (26.6%) for the average daily hours spent seated, None (58.9%) for engagement in physical activities lasting 1 h or more per day, and 1 day (56.0%) for the frequency of strength training sessions per week. When adolescents’ subjective health status was average, the most frequent responses were “13 h or more” (38.0%) for the average daily hours spent seated, None (68.0%) for engagement in physical activities lasting 1 h or more per day, and 1 day (66.0%) for the frequency of strength training sessions per week. When adolescents’ subjective health status was bad, the most frequent responses were 8 h or more (35.0%) for the average daily hours spent seated, None (80.0%) for engagement in physical activities lasting 1 h or more per day, and 1 day” (70.0%) for the frequency of strength training sessions per week.

**Table 3:** Correlation between adolescents’ subjective health status and physical activities.

Variable	N	Subjective health status			χ^2^ (*p*)
		Good	Average	Bad	
Average daily hours spent seated					
<8 h	64(15.3)	43(17.4)	14(9.3)	7(35.0)	16.857*
9–10 h	128(30.6)	73(29.4)	50(33.3)	5(25.0)	
11–12 h	99(23.7)	66(26.6)	29(19.4)	4(20.0)	
13 h or more	127(30.4)	66(26.6)	57(38.0)	4(20.0)	
Engagement in physical activities lasting 1 h or more per day					
No	264(63.2)	146(58.9)	102(68.0)	16(80.0)	5.908*
Yes	154(36.8)	102(41.1)	48(32.0)	4(20.0)	
Frequency of strength training sessions per week					
1 day	252(60.3)	139(56.0)	99(66.0)	14(70.0)	5.437
2–3 days	89(21.3)	56(22.6)	30(20.0)	3(15.0)	
4 days or more	77(18.4)	53(21.4)	21(14.0)	3(15.0)	
Total	418(100.0)	248(59.3)	150(35.9)	20(4.8)	

*p < 0.1, **p < 0.01, ***p < 0.001.

### 3.4 Influencing factors of adolescents’ subjective health status

The results of the analysis of factors affecting adolescents’ subjective health status are shown in Table 4. In Model 1, which considered socioeconomic factors and subjective health status, the frequency of breakfast consumption per week (*β* = 0.057, *p* = 0.008) exhibited a significant relationship with subjective health status. Specifically, a higher frequency of breakfast consumption was associated with better subjective health status. Model 2, which incorporated physical activity factors, reinforced this finding. A higher number of breakfasts consumed per week (*β* = 0.054, *p* = 0.012) was significantly related to subjective health status. In Model 3, which further included mental-health indicators, the frequency of breakfast consumption per week (*β* = 0.049, *p* = 0.024), and the presence or absence of suicidal ideation in the past year (*β* = −0.405, *p* = 0.046) were revealed to be correlated. More specifically, a higher frequency of breakfast consumption per week was associated with better subjective health status, while a reduction in suicidal ideation over the past year was also linked to better subjective health status.

**Table 4:** Influencing factors of adolescents’ subjective health status.

Variable	Model 1		Model 2		Model 3	
	*β*	*p*	*β*	*p*	*β*	*p*
Educational stage	−0.011	0.851	−0.013	0.820	0.003	0.980
Sex	−0.035	0.485	0.012	0.815	0.019	0.717
Income quintile	0.029	0.172	0.027	0.210	0.023	0.281
Health insurance type	−0.001	0.781	−0.001	0.862	−0.001	0.898
Subjective body shape recognition	−0.094	0.004	−0.097	0.003	−0.091	0.006
Sleep duration per week	0.014	0.738	0.016	0.695	0.012	0.779
Frequency of breakfast consumption per week	0.057	0.008	0.054	0.012	0.049	0.024
Average daily hours spent seated			0.101	0.330	0.102	0.409
Engagement in physical activities lasting 1 h or more per day			0.056	0.109	0.056	0.101
Frequency of strength training sessions per week			−0.002	0.964	0.052	0.980
Degree of stress recognition					−0.059	0.306
Prolonged depression for 2 weeks or more					0.125	0.298
Suicidal ideation in the past year					−0.405	0.046
Suicide planning in the past year					−0.153	0.666
Suicide attempts in the past year					−0.047	0.895
Counseling for mental-health problems in the past year					−0.112	0.459
	Adj *R*^2^ = 0.158, *F* = 2.648, *p* = 0.005		Adj *R*^2^ = 0.176, *F* = 2.642, *p* = 0.002		Adj *R*^2^ = 0.198, *F* = 2.270, *p* = 0.002	

## 4 Discussion

This study examined the relationship between self-rated health and mental health among Korean adolescents. While subjective health perception has been recognized as a strong predictor of general well-being and future health status (9, 10), existing research has primarily concentrated on adult and older adult populations (11–13). In South Korea, despite a high national life expectancy, only a small proportion of individuals consider themselves healthy, with adolescents reporting particularly low levels of life satisfaction and elevated symptoms of depression (8). These trends highlight the urgent need to understand adolescents’ perceived health status, not only as a reflection of their mental well-being but also as a foundation for early intervention and the development of tailored health policies.

An analysis of the correlation between general characteristics and adolescents’ subjective health status revealed significant associations with income quintile, degree of stress recognition, and frequency of breakfast consumption per week. Furthermore, adolescents in higher income brackets tended to report better subjective health.

Overall, 59.3% of adolescents perceived their subjective health status as good. This finding consistent with Kang (17), who reported that 62.8% of adolescents held positive health perceptions. While this perception may stem naturally from physical developments during adolescence, such as the development of secondary sexual characteristics, it highlights the need for public health programs to promote healthy daily habits that support adolescents in growing into healthy adults.

Regarding household income, adolescents from higher income brackets reported better subjective health, while those from lower income levels perceived their health less favorably. Specifically, higher income quintiles (annual household income of $157,723 or more, corresponding to the top 20% of U.S. income distribution in 2025) were associated with better self-rated health status. This finding is consistent with that reported by Prasad et al. (14) who observed a similar relationship between economic status and health perceptions among different demographic groups. The association between income and subjective health status underscores the significant impact of economic stability on overall well-being. Higher income levels often correlate with better access to healthcare, nutritious food, and health-promoting activities, which can contribute to more positive health perceptions. Conversely, lower-income households may encounter barriers to accessing these resources, potentially leading to less favorable health perceptions.

Health behaviors and subjective health status, which were measured using indicators such as sleep duration per week, frequency of breakfast consumption per week, exhibited significant differences. These findings align with those by Gentzler et al. (4), which indicated that a higher frequency of breakfast consumption per week was associated with more appropriate sleep duration per week. Consistently having breakfast and maintaining adequate sleep seem to create conditions that enable adolescents to focus on their studies, thereby enhancing academic efficiency. This improvement can, in turn, reduce potential sources of academic stress and mitigate negative effects on coping skills and interpersonal relationships, ultimately leading adolescents to perceive their health status more positively.

Analysis of the correlation between adolescents’ subjective health status and mental health, revealed notable associations with mental-health indicators, including stress recognition, suicidal ideation in the past year, suicide planning in the past year, suicide attempts in the past year, and mental-health counseling in the past year. Adolescents with a good subjective health status tended to report lower stress levels and absence of depression, whereas those with bad subjective health status experienced higher stress levels and depression. Studies reporting that individuals develop protective factors in response to perceived stress support these findings (23). High stress recognition often contributes to physical and mental-health disorders, feeding a cycle in which poor subjective health perception worsens, leading to increased depression and further deterioration in perceived health status. It is crucial for the government to reassess the roles and functions of local mental-health welfare centers. Through stronger partnerships with schools, creating a supportive social environment and accessible institutions where adolescents can seek help may prevent worsening trends in adolescent depression and suicide.

Adolescents’ subjective health status and their physical activity levels show a correlation. Specifically, the average daily hours spent seated and the extent of engagement in physical activities lasting 1 h or more per day were found to be associated with how adolescents perceive their health. This finding aligns with the results by Matsumoto et al. (24), who demonstrated that appropriate levels of exercise can help adolescents improve physical functioning and reduce depression, stress, and suicidal ideation. In the context of South Korean educational culture, where adolescents often spend extended hours seated in schools or private academies, a clear pattern emerges: the more time students spend seated, the more they perceive their subjective health status as bad. Conversely, students who engaged in at least 1 h of physical activity daily perceived their subjective health status as good. These findings highlight the importance of exploring school-level strategies within the South Korean educational framework to reduce prolonged seated activities and promote increased physical activity among adolescents.

When analyzing factors affecting adolescents’ mental health in relation to their subjective health status, the frequency of breakfast consumption per week and suicidal ideation in the past year were found to be influencing factors. This suggests that health behaviors and mental-health indicators play significant roles in adolescents’ perceptions of their health. Thus, developing school health programs that not only enhance physical health, but also foster mental and social well-being, enabling adolescents to grow into well-rounded, healthy adults, is necessary.

## 5 Limitations of the study and implications for research and practice

This study, utilizing secondary data from the Korea National Health and Nutrition Examination Survey (KNHNES), has some limitations. First, the cross-sectional design inherently limits causal inference between subjective health status and mental health, because temporal relationships cannot be established. Second, the exclusion of out-of-school adolescents restricts the generalizability of findings to all Korean adolescents, potentially overlooking unique challenges faced by this subgroup. Third, reliance on self-reported data for sensitive topics like mental health and health behaviors introduces risks of social desirability bias and subjective interpretation. Fourth, the KNHNES’s predefined variables constrained the analysis, because certain factors relevant to adolescent health (e.g., academic stress, social support networks) were not included. Finally, the cross-sectional nature of the survey precludes longitudinal assessment of health trajectories.

## 6 Conclusion

The findings revealed relationships among variables, such as subjective health status, demographic factors, health behaviors, and mental health, with notable effects linked to the frequency of breakfast consumption per week, and suicidal ideation in the past year. The results provide valuable insights for policymakers aiming to improve adolescent health by providing foundational data to address health inequalities and to inform strategies for physical and mental-health education at the school level. Future research would benefit from more detailed and quantifiable measures of the variables used in this study. This could improve the accuracy of sample characteristics and further support the validity of the study’s findings.

## Data Availability

Publicly available datasets were analyzed in this study. This data can be found at: http://gofile.me/3IxKZ/GpF0uNC1O. Further inquiries can be directed to the corresponding author.
